# Prevalence of Split Nerve Fiber Layer Bundles in Healthy People Imaged with Spectral Domain Optical Coherence Tomography

**DOI:** 10.4274/tjo.49358

**Published:** 2016-12-01

**Authors:** Sirel Gür Güngör, Ahmet Akman, Almila Sarıgül Sezenöz, Gülşah Tanrıaşıkı

**Affiliations:** 1 Başkent University Faculty of Medicine, Department of Ophthalmology, Ankara, Turkey

**Keywords:** Retinal nerve fiber layer, split nerve fiber layer bundles, optical coherence tomography

## Abstract

**Objectives:**

The presence of retinal nerve fiber layer (RNFL) split bundles was recently described in normal eyes scanned using scanning laser polarimetry and by histologic studies. Split bundles may resemble RNFL loss in healthy eyes. The aim of our study was to determine the prevalence of nerve fiber layer split bundles in healthy people.

**Materials and Methods:**

We imaged 718 eyes of 359 healthy persons with the spectral domain optical coherence tomography in this cross-sectional study. All eyes had intraocular pressure of 21 mmHg or less, normal appearance of the optic nerve head, and normal visual fields (Humphrey Field Analyzer 24-2 full threshold program). In our study, a bundle was defined as ‘split’ when there is localized defect not resembling a wedge defect in the RNFL deviation map with a symmetrically divided RNFL appearance on the RNFL thickness map. The classification was performed by two independent observers who used an identical set of reference examples to standardize the classification.

**Results:**

Inter-observer consensus was reached in all cases. Bilateral superior split bundles were seen in 19 cases (5.29%) and unilateral superior split was observed in 15 cases (4.16%). In 325 cases (90.52%) there was no split bundle.

**Conclusion:**

Split nerve fiber layer bundles, in contrast to single nerve fiber layer bundles, are not common findings in healthy eyes. In eyes with normal optic disc appearance, especially when a superior RNFL defect is observed in RNFL deviation map, the RNLF thickness map and graphs should also be examined for split nerve fiber layer bundles.

## INTRODUCTION

The retinal nerve fiber layer (RNFL) contains ganglion cell axons, which are one of the components of the data pathway from the retinal photoreceptors to the visual cortex in the brain. Studies using scanning laser polarimetry (SLP) have demonstrated that the axons originating from the optic disc form two bundles (superior and inferior). Nerve fiber bundles may diverge, but these split bundles are physiologic rather than pathologic.^1,2^

Pieroth et al.^3^ first described the ‘double hump’ pattern on optical coherence tomography (OCT) in individuals with split superior bundles. Colen and Lemij^2^ also described superior, inferior or both split bundle patterns as SLP imaging findings.

The aim of this study was to describe split nerve fiber layer bundles and determine their prevalence in the healthy population of Turkey.

## MATERIALS AND METHODS

The data of 359 subjects were examined cross-sectionally. The mean age was 43.5±8.8 years (range, 30-60 years). All subjects had intraocular pressure under 21 mmHg, normal visual field (on Humphrey Visual Field test 24-2 full threshold program) and normal optic nerve head. None of the subjects had any systemic or ocular diseases.

Seven hundred eighteen eyes of 359 healthy subjects were examined by Cirrus HD spectral-domain OCT (Carl Zeiss Meditec, Dublin, CA, USA) under mydriasis. Subjects with signal strength less than 6 were not included in the study. In our study, we defined ‘split bundles’ as those showing a localized defect not resembling a wedge defect on the RNFL deviation map together with a relatively symmetrically division on the thickness map. Classification was performed by two independent observers. To standardize the classification, both observers used the reference example sets created by Colen and Lemij^2^ The final groups were formed by consensus agreement between the two observers for all subjects.

In fact, there is a wide spectrum between a fully split bundle and a single bundle, and partially split bundles have been previously described.^2^ As a working principle, our criteria for a fully split bundle was that the nerve fiber bundle diverged completely extending to the optic nerve head on the RNFL thickness map and this divergence was reflected on the thickness graph as a double hump. We only included fully split bundles in this study.

## RESULTS

**Figure 1:**
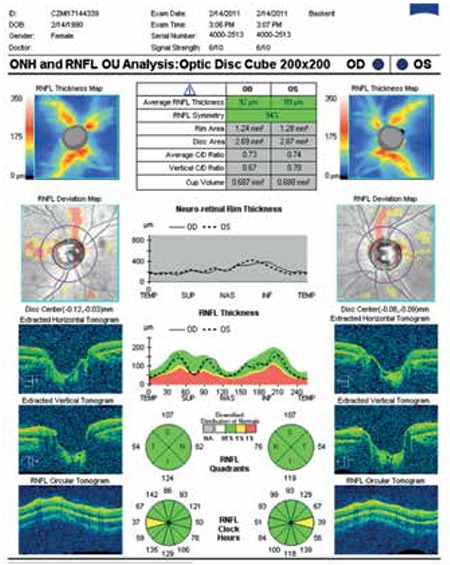
Bilateral split superior retinal nerve fiber bundle

**Figure 2:**
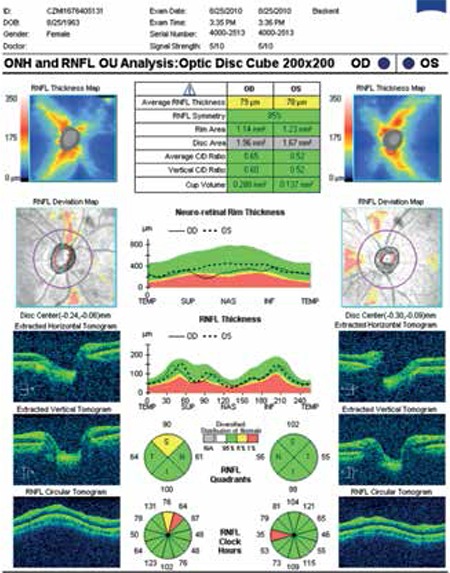
Right split superior retinal nerve fiber bundle

The interobserver agreement of the classification was evaluated using ĸ statistics, and the ĸ value of 0.85 showed good agreement. In cases of disagreement, a consensus was reached. Nineteen subjects (5.29%) had bilateral superior split bundle (Figure 1) and 15 (4.16%) had unilateral superior split bundle (Figure 2). Split bundles were not detected in the remaining 325 subjects (90.52%). The distribution of split retinal nerve fiber bundles in our subjects is shown in Table 1.

Of the subjects with unilateral split superior bundles, 8 (2.23%) were in right eyes and 7 (1.95%) were in left eyes. The prevalence of split bundles in right and left eyes was comparable (p=0.67).

## DISCUSSION

In healthy eyes, the peripapillary RNFL surrounding the optic disc is thickest in the superior and inferior quadrants and thinner in the nasal and temporal quadrants, exhibiting a double-hump pattern on the temporal-superior-nasal-inferior-temporal (TSNIT) graph.^4,5^ Pieroth et al.^3^ first described the split bundle pattern on OCT in a healthy eye. Colen and Lemij^2^ demonstrated with GDx fixed corneal compensation data that a proportion of normal eyes exhibited superior and inferior split bundles on SLP, resulting in a triple or quadruple hump pattern in the RNFL thickness modulation graph. In other words, the superior and inferior bundles have two peaks. Of 454 eyes of 254 healthy subjects, Colen and Lemij^2^ observed a clear split superior bundle pattern in 6.4%, clear split inferior bundle pattern in 1.1%, and both split superior and inferior bundle patterns in 0.2% of the eyes. We also observed split superior bundles in 9.18% of the subjects in our study, similar to the results of Colen and Lemij.^2^

Using GDx variable corneal compensation, Kaliner et al.^6^ demonstrated in a healthy eye with superior split bundle that the division in the bundle became more pronounced as the diameter of the measurement ring surrounding the optic disc increased. As a continuation of this study, Kaliner et al.^6^ did a histologic investigation to determine whether the split bundle pattern was a real phenomenon. They performed a post mortem examination of 14 eyes of 13 patients and found the prevalence of split bundles was 36% (5/14; 3 superior, 2 inferior). None of the eyes exhibited both split superior and split inferior bundles. The high prevalence of split bundles found in this study compared to others can likely be attributed to low patient number. That study definitively demonstrated that split bundles are not an artifact of RNFL imaging but instead a real anatomic finding.

Glaucoma is a chronic optic neuropathy characterized by progressive optic nerve damage and typical visual field losses due to retinal ganglion cell death. Methods that provide reliable and objective data regarding optic disc and RNFL damage are critical in the diagnosis and monitoring of glaucoma. The use of OCT has become increasingly common to measure RNFL thickness and optic nerve head parameters in the diagnosis and management of glaucoma.^7^ Although split bundles appear to be a normal finding that does not indicate disease, they may affect GDx and OCT parameters and be mistaken for wedge defect. In contrast to split bundles, wedge defects are separated from adjacent tissue with sharper margins and occur with glaucomatous changes in the optic nerve.^2^ In the deviation maps and quadrant or clock hour graphs of certain imaging modalities, split bundles may give the impression of decreased retinal nerve fiber thickness compared to the normative data. In fact, TSNIT analysis without evaluating physiological variance between bundles within normative values is not very sensitive or specific. Determining separate normative value ranges for split and single bundles may increase sensitivity.

## CONCLUSION

Split nerve fiber bundles may be encountered in healthy eyes. For individuals with a normal, healthy optic nerve on examination, the RNFL thickness map and graph should be assessed for split nerve fiber bundles, especially in the presence of a superior RNFL defect on the RNFL deviation map.

### Ethics

Ethics Committee Approval: Retrospective study. Informed Consent: It was taken.

Peer-review: Externally peer-reviewed.

## Figures and Tables

**Table 1:**
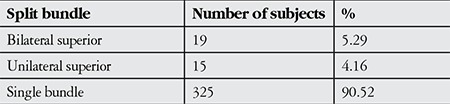
Distribution of split retinal nerve fiber bundles in the study subjects
